# Caffeic acid phenethyl ester attenuates *Enterococcus faecalis* infection in vivo: antioxidants and NF-κB have a protective role against stomach damage

**DOI:** 10.25122/jml-2023-0544

**Published:** 2024-06

**Authors:** Abdulaziz Yahya Al-Ghamdi

**Affiliations:** 1 Biology Department, Faculty of Science, Al-Baha University, Al-Baha, Saudi Arabia

**Keywords:** *Enterococcus faecalis*, CAPE, NF-κB, PCNA, antioxidant

## Abstract

The mammalian gastrointestinal tract hosts a significant microbial symbiont community, an intriguing feature of this complex organ system. This study aimed to investigate the anti-inflammatory, antioxidant, and protective effects of caffeic acid phenethyl ester (CAPE) against *Enterococcus faecalis* infection in the stomach at a dose of 10^6^ CFU in Swiss mice. A total of 30 mice were randomly assigned to three groups of ten mice each. Group I was the negative control, Group II was infected orally with *E. faecalis* for 18 days, and Group III was infected with *E. faecalis* and treated with CAPE orally at a daily dose of 4 mg/kg for 18 days. We assessed the antioxidant activities of stomach homogenate and the immunohistochemical expressions of the transcription factor nuclear factor kappa B (NF-κB) and proliferating cell nuclear antigen (PCNA). Histopathological examination was performed on the stomachs of all mice. Group II had decreased levels of antioxidant activity and positive expressions of NF-κB and PCNA. Histological observations revealed an increase in mucosal and glandular thickness compared with Group I. Group III, treated with CAPE, showed a significant increase in antioxidant activities and a significant decrease in NF-κB and PCNA immunoreactivities compared with Group II. In addition, Group III showed restoration of the normal thickness of the non-glandular and glandular parts of the stomach. Our results revealed that *E. faecalis* infection has damaging effects on the stomach and proved that CAPE has promising protective, anti-inflammatory, and antioxidant effects against *E. faecalis*. Further studies may investigate the potential therapeutic effects of CAPE against *E. faecalis* infection.

## INTRODUCTION

*Enterococcus faecalis* is a Gram-positive, facultative anaerobic bacterium found in various environments, including the gastrointestinal tract of humans and animals, soil, water, and fermented foods [1,2]. Despite its commensal nature, *E. faecalis* is responsible for many infections, such as urinary tract infections, endocarditis, and surgical site infections [3]. Its adaptability, intrinsic resistance to antibiotics, and ability to acquire exogenous resistance genes through horizontal gene transfer pose significant challenges to its clinical management [4,5].

*E. faecalis* is a leading cause of nosocomial infections, particularly in immunocompromised patients or those undergoing invasive procedures. The development of multidrug-resistant strains further complicates treatment options, necessitating a multifaceted approach to managing *E. faecalis* infections [6,7]. Owing to its intrinsic and acquired resistance mechanisms, *E. faecalis* has become less susceptible to many commonly used antibiotics, including β-lactams, aminoglycosides, and vancomycin [8]. Therefore, a deeper understanding of *E. faecalis* and the development of novel therapeutic strategies are needed to improve patient outcomes [9,10].

An important active ingredient in honeybee propolis extract, caffeic acid phenethyl ester (CAPE) has long been used in traditional medicine [11]. Research has demonstrated that CAPE exhibits a range of beneficial properties, including anti-inflammatory, immunomodulatory, antineoplastic, antioxidant, and wound-healing effects [12,13]. The inflammatory response is initiated by releasing chemical mediators from damaged tissues and migrating cells. These mediators include biogenic amines, eicosanoids (metabolites of arachidonic acid), platelet-activating factors, cytokines like interleukins and tumor necrosis factor-α (TNF-α), and reactive oxygen species [14]. These compounds are produced by inflammatory cells, including mast cells, endothelial cells, macrophages, monocytes, lymphocytes, and polymorphonuclear leukocytes (neutrophils, eosinophils, and basophils) [15]. CAPE suppresses the inflammatory process by preventing the production of chemokines, cytokines, T-cell proliferation, and lymphokines. In particular, CAPE is a strong and selective inhibitor of activating the transcription factor nuclear factor kappa B (NF-κB), which may be the molecular basis for its various anti-inflammatory and immunomodulatory effects [16,17].

NF-κB is involved in numerous physiological processes, such as inflammation, cell division, and immunological responses [18]. It facilitates the transcription of several cytokines, enzymes, chemokines, antiapoptotic factors, and cell growth factors [19]. Both in vitro and in vivo studies have shown that CAPE, at micromolar concentrations, exhibits various biological activities, including the selective inhibition of NF-κB and the suppression of the lipoxygenase pathway of arachidonic acid metabolism during inflammation [14]. CAPE inhibits NF-κB activation triggered by reactive oxygen species (ROS)-generating agents in human histiocytic and coronary artery endothelial cells [20]. It achieves this by blocking the interaction between NF-κB proteins and DNA rather than preventing the degradation of inhibitor κB α. [21]. The inhibition of ROS suppression of NF-κB activation and the direct inhibition of iNOS catalytic activity are most likely caused by the anti-inflammatory effect of CAPE [22].

This study aimed to highlight the anti-inflammatory and protective role of CAPE against acute inflammation caused by *E. faecalis*. We assessed various inflammatory and biochemical markers in stomach tissue homogenates, including antioxidants and enzymes. It also aimed to analyze NF-κB, with a specific focus on proliferating cell nuclear antigen (PCNA) as an important inflammatory marker. This study may help diagnose and monitor inflammatory conditions.

## MATERIAL AND METHODS

### Animals

Healthy adult Swiss albino male mice (22–25 g) were obtained from King Abdulaziz University, Jeddah, Saudi Arabia. Mice were acclimatized for 5 days at the Faculty of Science, Al-Baha University, under adapted temperature, kept in a 12-h light-dark cycle, and maintained on a standard diet and water ad libitum. The study followed the animal handling guidelines of the Ethical Committee and Scientific Research, University of Al-Baha.

### Enterococcus faecalis

Ten mice were used to determine the number of viable *Enterococcus faecalis* bacteria. We performed gastric gavage with 0.5 ml of diluent from a frozen overnight culture, using a Perfektum stainless-steel feeding tube (Popper & Sons), aiming to inoculate each mouse with 10^6^ colony-forming units (CFUs). The weight and clinical condition of the mice were monitored every 2–3 days. Each mouse received *E. faecalis* (10^6^ CFU/mouse) three times a week for three weeks. The number of viable *E. faecalis* bacteria was determined in mouse feces using the drop plate method [23]. Fecal samples were dissolved in sterile phosphate-buffered saline (PBS), and 10 µL of each serial dilution was plated three times as drops onto Enterococcagar to detect *E. faecalis*. Petri dishes were incubated aerobically at 37 °C for two days. Colonies displaying typical morphology on Enterococcagar differential media were counted, averaged, and multiplied by the dilution factor, accounting for the weight of the fecal sample.

### CAPE and lethality study

To create a solution with a 15 mg/ml concentration, CAPE was dissolved in a droplet of dimethyl sulfoxide (DMSO) (Sigma). According to their intended usage, different substance concentrations were made in PBS. The mice were divided into four groups of ten each and were given serialized CAPE concentrations. The number of live mice was counted each day. The procedure was continued following ethical standards for 30 days. Following the injection of DMSO, a series of ten mice tests showed no significant effect, and the vehicle was considered appropriate for administration.

### Experimental design

Mice were randomly distributed into three groups of ten mice per group. Group I was kept as the negative control. Group II was injected orally with *E. faecalis* at a dose of 10^6^ CFU in 0.5 ml of saline every 2 days for 18 days and did not receive any treatment. Group III was injected orally with *E. faecalis* at a dose of 10^6^ CFU in 0.5 ml of saline and treated orally daily with 4 mg/kg of CAPE, starting from day 1, concurrent with *E. faecalis* 10^6^ CFU in 0.5 ml of saline, for 18 days, after a high level of stool colonization in mice was established. At 24 h after the last treatment, the stomach was removed, cleaned, and prepared to assess the levels of antioxidants superoxide dismutase (SOD), glutathione-S-transferase (GST), glutathione peroxidase (GSH-Px), and malondialdehyde (MDA). The remaining stomach was kept in 10% formalin for immunohistochemical and histopathological assessment.

### Determination of SOD, GST, GSH-Px, and MDA activity

The stomach tissue was cut into small pieces, washed with PBS, and ground in a homogenization buffer (0.05 M Tris-HCl pH 7.9, 25% glycerol, 0.1 mM EDTA, and 0.32 M (NH_4_)_2_SO_4_) containing a protease inhibitor tablet (Roche). The tissue lysates were then homogenized on ice using an HG-15D homogenizer (Witeg Labortechnik). The solution was sonicated for 15 seconds in an ice bath to avoid overheating. Next, it was centrifuged for 5 min at 12,000 r.p.m. and 4 °C. After isolating the supernatant and storing it at −80 °C, the homogenate’s SOD, GST, and GSH-Px activity was measured by determining the amount of protein in each sample. GST activity was assessed by reacting the GSH−SH group with 1-chloro-2,4-dinitrobenzene, while GSH-Px activity was measured by monitoring the reduction of t-butyl hydroperoxide with nicotinamide adenine dinucleotide phosphate. All measurements were performed using a Spectro 24RS visible spectrophotometer (Labomed) with a temperature-controlled cuvette holder at 19 °C, and enzyme activities were expressed in U/g. The following wavelengths were used in the analysis: 480 nm for SOD, 340 nm for GST, and 412 nm for GSH-Px, as described previously [24]. Malondialdehyde (MDA) is a primary byproduct of lipid peroxidation. It reacts with thiobarbituric acid, along with other byproducts, to form a colored complex that has a maximum absorbance of 535 nm. This absorbance represents the color produced by all thiobarbituric acid-reactive substances [25].

### Immunohistochemical examination of NF-κB

Immunohistochemistry was used to assess the expression of NF-κB in all mouse groups. After rinsing in PBS, the prepared sections were covered for 1.5 h in 1: 100 mouse anti-NF-κB p65 antibodies (Sigma-Aldrich). The sections were then washed and treated for 30 min with a secondary antibody tagged with a poly-horseradish peroxidase enzyme (Sigma-Aldrich). The slides were treated with freshly prepared 3,3′ diaminobenzidine tetrahydrochloride solution for 5 min, washed and treated with Mayer’s hematoxylin, dehydrated in graded alcohol (50%, 70%, 90%, and 100%), cleared with xylene, and mounted using a nonaqueous permanent mounting medium, as described previously [26].

### Immunohistochemical examination of PCNA

Each mouse stomach was removed, and 4mm slices were cut and put on silane-coated slides. The sections were deparaffinized with 100% xylene and rehydrated through a graded ethanol series. For antigen retrieval, the sections were immersed in tris-buffered saline (TBS) with a pH of 6 and heated in a microwave oven at 750 W. After cooling to room temperature, the sections were treated with MCM7 monoclonal mouse anti-human primary antibodies (Thermo Fisher Scientific) and PCNA monoclonal mouse anti-human antibodies (Thermo Fisher Scientific) at a dilution of 1:2.000 for 1 h. Then, the sections were treated with Dako Envision following washing in TBS. Antibody expression was visualized using diaminobenzidine and counterstained with Mayer’s hematoxylin [27].

### Histopathological examination of the stomach

The remaining stomach tissue was sectioned at 5 µm thickness after being dried, fixed in paraffin, and preserved in 10% paraformaldehyde. The sections were stained with hematoxylin and eosin (H&E). Morphological changes were assessed under an Eclipse 80i microscope (Nikon), and images were recorded using a DS-Fi1 digital microscope camera (Nikon).

### Sample size

Sample size calculation was based on previous equivalent studies [28]. Using G*power v.3.1.9.5 to calculate the sample size based on an effect size of 1.6254, a two-tailed test, an α error of 0.05, and a power of 90.0%, the sample size was determined as ten in each group.

### Computer-assisted digital image analysis (digital morphometric study)

Slides were photographed using an MVV5000CL digital eyepiece with a 5.0 M pixel sensor installed on a MEIJI MX5200L microscope using 20× and 40× objectives. The resulting 20× images were analyzed on an Intel Core i7-based computer using Fiji ImageJ v.1.51r (NIH). To measure the percentage of the staining surface area, the color deconvolution 2 plugin was used. Five random fields from each tissue specimen were analyzed, as described previously [29].

### Statistical analysis

Data were analyzed using GraphPad Prism 9 (GraphPad Software). Numerical data were analyzed for normality using the Shapiro–Wilk test and presented as mean ± SD. One-way analysis of variance (ANOVA) and Tukey’s test were used to compare parametric data. A *P* value of ≤0.05 was considered statistically significant.

## RESULTS

### Enterococcus faecalis infection and the effects of CAPE on the activities of SOD, GST, GSH-Px, and MDA

To assess the impact of *E. faecalis* infection, we measured the activities of superoxide dismutase (SOD), glutathione S-transferase (GST), glutathione peroxidase (GSH-Px), and malondialdehyde (MDA) in the stomach tissues. Group II, infected with *E. faecalis*, showed significantly decreased levels of SOD, GST, and GSH-Px compared to the control Group I. The significant changes observed in the parameters were dose-dependent at 10^6^ *E. faecalis* inoculated orally. SOD levels were 36.17 ± 2.87 U/g in Group II versus 67.49 ± 3.18 U/g in Group I (*P* < 0.001). GST and GSH-Px levels in Group II were 37.11 ± 2.67 U/g and 36.13 ± 1.41 U/g, respectively, compared to 76.50 ± 1.98 U/g and 75.59 ± 1.67 U/g in Group I (*P* < 0.001). Conversely, MDA levels were significantly higher in Group II (15.92 ± 0.42 nmol/mg) compared to Group I (9.47 ± 0.32 nmol/mg; *P* < 0.001). The findings showed that oral treatment with CAPE at a dose of 4 mg/kg in Group III elicited a significant increase in SOD, GST, and GSH-Px levels and a decrease in MDA, especially when compared with Group II (*P* < 0.001). Thus, treatment with CAPE at the chosen dose of 4 mg/kg enhanced the elimination of ROS in the mouse stomach (Table 1).

**Table 1:** SOD, GST, GSH, and MDA levels across different groups

Parameter	Group I (control)	Group II (infected with *E. faecalis*)	Group III (infected with E. faecalis and treated with CAPE	*P* value
**SOD (U/g)**	67.49 ± 3.18	36.17 ± 2.87^***^	66.84 ± 3.82 *^###^*	<0.0001
**GST (U/g)**	76.50 ± 1.98	37.11 ± 2.67^***^	74.78 ± 1.19^###^	<0.0001
**GSH (U/g)**	75.59 ± 1.67	36.13 ± 1.41^***^	75.10 ± 1.38^###^	<0.0001
**MDA (U/g)**	9.47 ± 0.32	15.92 ± 0.42^***^	9.89 ± 0.89^###^	<0.0001

Data expressed as mean ± SD. One-way ANOVA followed by post-hoc Tukey’s test. *P < 0.05 vs. Group I. #P < 0.05 vs. Group II.

### Immunohistochemical expression of NF-κB

**Figure 1:**
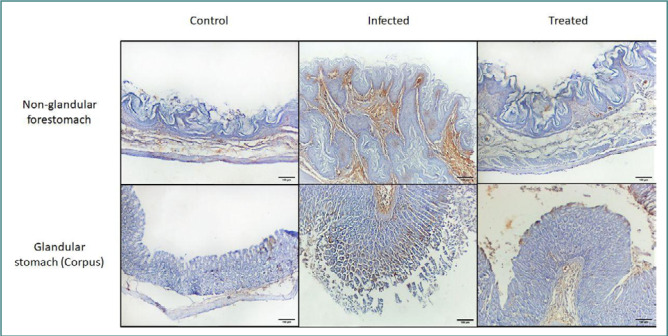
Immunohistochemical staining of NF-κB among mice in different study groups, in the non-glandular forestomach (upper row) and glandular stomach (lower row). The control group shows minimal to no NF-κB immune reactivity in the non-glandular and glandular parts. Group II, infected with *Enterococcus faecalis* at a dose of 10^6^ CFU shows increased NF-κB immune reactivity in both stomach parts. Group III, treated with CAPE, shows a marked reduction in NF-κB immune reactivity in the non-glandular and glandular parts of the stomach. Original magnification 100×.

Regarding the immunohistochemical expression of NF-κB, stained sections of stomachs from Group I exhibited normal appearance, with a negative expression of NF-κB in the non-glandular and glandular parts. In Group II, the stomach showed increased expression of NF-κB in the non-glandular and glandular parts, whereas, in Group III, there was a marked reduction in NF-κB immune reactivity in the non-glandular and glandular parts of the stomach (Figure 1). We observed a significant increase in the size of the NF-κB-positive area in Group II compared to the control group and a significant decrease in the size of the NF-κB-positive area in Group III compared to Group II (Table 2).

**Table 2:** Comparison of NF-κB immunohistochemical staining among mice in different groups. Data expressed as percentage of NF-κB-positive area.

Stomach part	Group I	Group II	Group III	*P* value
**Non-glandular**	2.41 ± 1.69	19.71 ± 7.57^*^	5.58 ± 2.65^#^	0.0002
**Glandular**	2.66 ± 3.55	18.97 ± 6.82^*^	6.19 ± 5.76^#^	0.0014

Data expressed as mean ± SD. One-way ANOVA followed by post-hoc Tukey’s test. *P < 0.05 vs. Group I. #P < 0.05 vs. Group II.

### PCNA immunoreactivity examination

**Figure 2:**
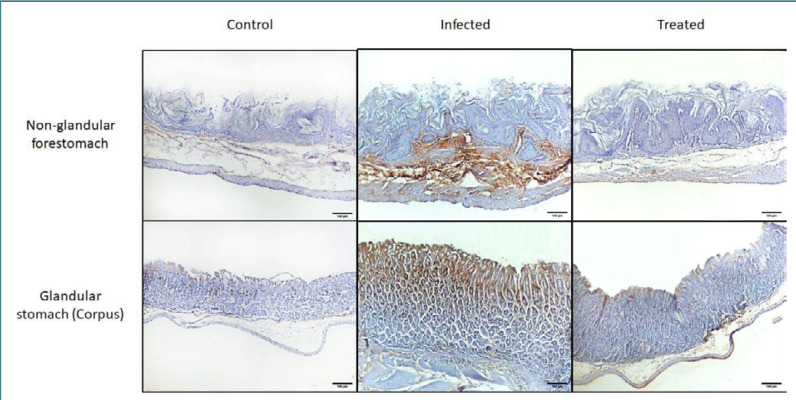
Immunohistochemical staining of PCNA among mice in different study groups, in the non-glandular forestomach (upper row) and glandular stomach (lower row). The control group shows minimal to no PCNA immune reactivity in the non-glandular and glandular parts. Group II, infected with *Enterococcus faecalis* at a dose of 10^6^ CFU shows increased PCNA immune reactivity in both stomach parts. Group III, treated with CAPE, shows a marked reduction in PCNA immune reactivity in the non-glandular and glandular parts of the stomach. Original magnification 100×.

**Figure 3:**
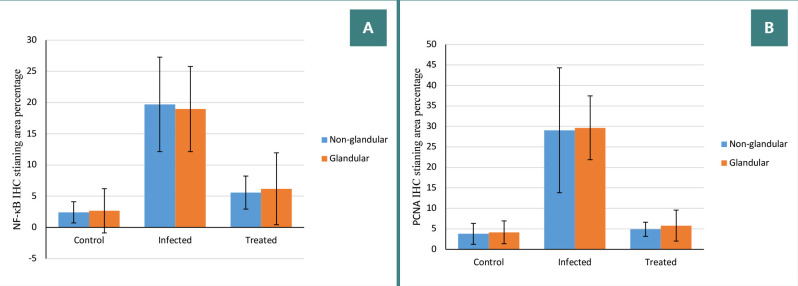
Comparison of NF-κB and PCNA immunohistochemical staining among mice in different groups. A, NF-κB IHC staining area percentage. B, PCNA IHC staining area percentage.

The immunohistochemical expression of PCNA showed no immunoreactivity in the control group. In Group II, there was significant immunoreactivity in both stomach parts, and in Group III, mild immunoreactivity was observed in the non-glandular and glandular parts of the stomach (Figures 2 and 3). There was a significant increase in the PCNA-positive area in Group II compared with the control group and a significant decrease in the PCNA-positive area in Group III compared with Group II (Table 3).

**Table 3:** Comparison of PCNA immunohistochemical staining among mice in different groups. Data expressed as percentage of PCNA-positive area.

Stomach part	Group I	Group II	Group III	*P* value
**Non-glandular**	3.78 ± 2.56	29.07 ± 15.24^*^	4.89 ± 1.72^#^	0.0011
**Glandular**	4.15 ± 2.79	29.66 ± 7.79^*^	5.80 ± 3.78^#^	<0.0001

Data expressed as mean ± SD. One-way ANOVA followed by post-hoc Tukey’s test. ^*^P < 0.05 vs. Group I. #P < 0.05 vs. Group II.

### Histopathological results (light microscopy examination of H&E-stained sections)

**Figure 4:**
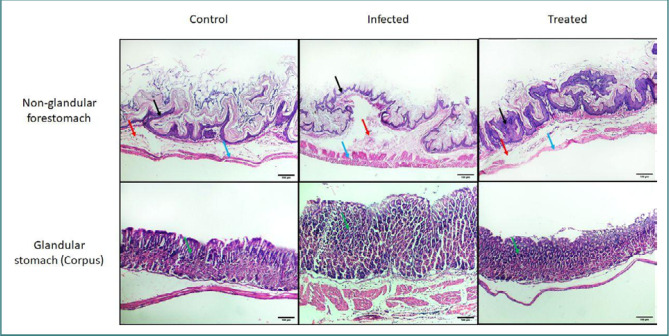
Histopathological examination (H&E staining) among mice in different study groups, in the non-glandular forestomach (upper row) and glandular stomach (lower row). The control group shows normal histological structure of stratified squamous keratinized epithelium with underlying submucosa and muscularis in the non-glandular part, and the fundus shows normal histological structure of gastric glands in the glandular part. Group II, infected with *Enterococcus faecalis* at a dose of 10^6^ CFU, shows increased mucosal and glandular thickness. Group III, treated with CAPE, shows almost complete restoration of the normal thickness of the non-glandular and glandular parts of the stomach. Black arrow, epithelium; red arrow, submucosa; blue arrow, muscles; green arrow, gastric glands. Original magnification 100×.

The H&E-stained stomach sections from the control group showed normal histological structure characterized by a stratified squamous keratinized epithelium, with an underlying submucosa and muscularis in the non-glandular part and a fundus showing normal histological structure of the gastric glands in the glandular part. In contrast, Group II exhibited increased mucosal and glandular thickness compared to the control. However, Group III had nearly complete restoration of normal thickness in both the non-glandular and glandular parts of the stomach (Figure 4).

## DISCUSSION

The human microbiome refers to the vast array of microorganisms that inhabit different body cavities, ranging from the skin and lungs to the vagina and, most prominently, the gastrointestinal tract. The gut microbiota, in particular, is recognized for its extensive diversity and abundance of microbial species [30]. Changes in the gut microbiota composition have been implicated in various diseases, including inflammatory bowel disease and colorectal cancer [31]. Therefore, an analysis to clarify the role of the microbiota in each stage of these diseases is required. New diagnostic techniques and potential therapies can be developed in response to changes in the microbiota of the gastrointestinal tract.

*E. faecalis* is an opportunistic pathogen that can translocate across the mucosal barrier to cause systemic infections [32]. It is primarily described as a core commensal member of the human gut, present in more than 90% of bacterial isolates [33]. In this study, *E. faecalis* administered orally at a dose of 10^6^ CFU resulted in a significant reduction of the antioxidant activity of SOD, GST, and GSH-Px compared with the control group (Table 1). Oxygen is abundant in Earth's atmosphere and plays a critical role in supporting diverse life forms. However, as pointed out by Markkanen [34], excessive reactive oxygen species (ROS) can chemically modify various macromolecules like RNA, DNA, proteins, and lipids through oxidation. As a result, the structure and function of these macromolecules are affected, leading to cell toxicity. According to Fasnacht and Polacek [35], ROS can be produced in bacteria and other organisms either internally due to aerobic metabolism or externally due to local exposure to elevated levels of oxidative agents. Cells can withstand low concentrations of ROS and have systems in place to combat oxidation.

According to Dryden *et al*., at low concentrations, ROS function as signaling molecules in regulating a variety of cellular functions, including quorum sensing, biofilm formation, and bacterial self-destruction [36]. Oxidative stress occurs when there is an imbalance between the quantity of ROS and the body’s capacity to remove them. This has been demonstrated by our results, which are consistent with those of Berghoff and Klug [37]. Three naturally occurring ROS species, namely superoxide anion (O_2_^−^), hydrogen peroxide (H_2_O_2_), and hydroxyl radical (HO^•^), show different reactivities and have major relevance in aerobic environments and oxidative stress, leading to decreased SOD, GST, and GSH-Px levels and increased MDA levels, as demonstrated by the results in Group II. These enzymes are balanced with ROS and occasionally inhibit the elevated levels of ROS. Moreover, Huycke *et al*. [38] found that *E. faecalis* produces substantial quantities of extracellular superoxide (O_2_^−^) and derivative ROS, such as H_2_O_2_ and hydroxyl radicals, through the autoxidation of membrane-associated demethylmenaquinone. These oxidants may have an important role in decreasing antioxidant levels, as suggested by the results of this study.

In contrast, Group III, which was infected with *E. faecalis* and treated with CAPE at a dose of 4 mg/kg, showed increased antioxidant enzyme expression and significantly improved SOD, GST, and GSH-Px levels compared with Group II (Table 1), with levels close to normal. These results are consistent with those of Pérez *et al*. [39]. They are also in accordance with the studies of Wang *et al*., Chen *et al*., and Wang *et al*. [40-42], which demonstrated the strong antioxidant properties of CAPE by quenching the 2,2-diphenyl-1-picrylhydrazyl radical and lowering the formation of the superoxide anion that results from the autoxidation of β-mercaptoethanol. In addition to its strong cytoprotective and antigenotoxic antilipoperoxidative potential against oxidative damage, CAPE also inhibits the activity of xanthine oxidase.

A growing body of research indicates that NF-κB is essential for controlling inflammation and the immune system, both of which are involved in the etiology of neurodegenerative diseases [43]. Pro-inflammatory and anti-inflammatory cytokines are released as a result of the innate and adaptive immune systems being activated during the inflammatory response. These cytokines are important for the resolution of inflammation [44]. The parenchyma of the brain also exhibits these systemic inflammatory reactions, also referred to as neuroinflammation. Patients with various neurodegenerative disorders, such as Alzheimer’s disease, Parkinson’s disease, amyotrophic lateral sclerosis, and frontotemporal dementia, have specific inflammatory signatures in their brains and bloodstream [45]. Interestingly, CAPE may be a relevant therapeutic agent for controlling NF-κB signaling and reducing neuroinflammation in these disorders [22].

In this study, Group II exhibited increased NF-κB immune reactivity, whereas the CAPE-treated Group III showed a significant reduction in NF-κB immune reactivity in the non-glandular and glandular parts of the stomach. The control group showed minimal to no NF-κB immune reactivity (Figure 1 and Table 2). Similar results were obtained by Zou and Shankar [46], who found that *E. faecalis* with macrophages instead of internalized bacteria had an important role in NF-κB activation and subsequent cytokine expression. This implies that the cell surface has a major role in the pathogen-associated molecular patterns phagocytes identify during enterococcal infections. The results of Group III are also consistent with those of Natarajan *et al*., Lee *et al*., and Sun *et al*. [47-49], who analyzed for the first time the molecular mechanisms through which CAPE prevents NF-κB activation.

CAPE has been found to completely block the activation of NF-κB through TNF-α in a dose- and time-dependent manner. Moreover, CAPE prevents NF-κB activation caused by okadaic acid, ceramide, hydrogen peroxide, phorbol ester, and other inflammatory substances. Reduction agents, on the other hand, reverse the inhibitory effects of CAPE, suggesting that sulfhydryl groups have a critical role in NF-κB activation. Moreover, CAPE inhibits the p65 subunit of NF-κB from moving to the nucleus, but it has no discernible effect on the TNF-α-induced degradation of IκBα. However, it delays IκBα resynthesis. Therefore, our findings support the hypothesis that CAPE inhibits p65 translocation and IκBα degradation in human chondrocytes to prevent NF-κB activation. PCNA, which is involved in DNA replication and repair, is an indicator of DNA damage [22].

Our finding that Group II (infected with *E. faecalis*) showed significant PCNA immunoreactivity is consistent with that of Kaplan *et al*. [50], who found that the increased detection of PCNA could be a secondary effect of cytokines originating from both inflammatory cells and keratinocytes. Thus, the immunodetection of PCNA by current immunohistochemical methods in the non-glandular and glandular parts of the stomach was sufficient to be used as an indicator of infection.

In contrast, in the CAPE-treated group, the histochemical and immunohistochemical analysis revealed that CAPE treatment significantly reduced the number of PCNA-positive cells, consistent with the results of Mir *et al*. [51], who found that CAPE led to the inhibition of PCNA expression in glioma cell proliferation and could prove to be an effective adjunct to therapies.

Lastly, the histopathological examination of stomachs was conducted across different study groups. The fact that Group II had an increase in mucosal and glandular thickness and Group III had an almost comprehensive restoration of the normal thickness of the non-glandular and glandular parts of the stomach demonstrates the potent effect of CAPE in protecting the stomach against infection, damage, and inflammation induced by *E. faecalis*.

## CONCLUSION

The results of this study suggest that CAPE has protective effects against *E. faecalis* infection of the stomach and that it could be potentially used in controlling inflammation and protecting from stomach injury in experimental models, as evidenced by its ability to increase the levels of antioxidant markers and reduce the inflammatory mediator NF-κB. The results also suggest that, alongside other markers, PCNA may serve as a promising diagnostic and prognostic marker in future assessments. Finally, these results highlight the diverse biological and pharmacological properties of CAPE, its capability to be a functional coadjuvant for preventing infection-mediated inflammation or gastrointestinal diseases, and its potential applications in human clinical trials as an antibacterial agent. Although these results shed some light on the processes underlying the benefits of CAPE, further research of the underlying molecular pathways and signaling cascades will improve our comprehension of the compound’s anti-inflammatory and protective qualities.

## Data Availability

Further data are available from the author upon reasonable request.

## Personal thanks

I would like to acknowledge the assistance of Dr. O.A. Elkashty at the Faculty of Dentistry, Mansoura University, in the histopathological part of this study.
